# Chair-Based Magnetic Pelvic Floor Stimulation and Female Sexual Function in Women with Urinary Incontinence: A Systematic Review

**DOI:** 10.3390/jcm14238496

**Published:** 2025-11-30

**Authors:** Geanina Sacarin, Marius Craina, Bogdan Sorop, Mihai Calin Bica, Lavinia Stelea, Mihaela Prodan, Madalina Sorop, Alina Simona Abu-Awwad, Maria Sorop-Florea, Adina Ruta, Razvan Nitu

**Affiliations:** 1 Doctoral School, Victor Babes University of Medicine and Pharmacy, 300041 Timisoara, Romania; geanina.sacarin@umft.ro (G.S.); madalina.pop@umft.ro (M.S.); 2 Department of Obstetrics and Gynecology, “Victor Babes” University of Medicine and Pharmacy, 300041 Timisoara, Romania; craina.marius@umft.ro (M.C.); stelea.lavinia@umft.ro (L.S.); alina.abuawwad@umft.ro (A.S.A.-A.); nitu.dumitru@umft.ro (R.N.); 3 Discipline of Clinical Abilities, Victor Babes University of Medicine and Pharmacy, 300041 Timisoara, Romania; mihaela.prodan@umft.ro; 4 Department of Obstetrics and Gynecology, Faculty of Medicine, University of Medicine and Pharmacy of Craioiva, 200349 Craiova, Romania; drfloreamm@gmail.com (M.S.-F.); ruta.adina92@gmail.com (A.R.)

**Keywords:** urinary incontinence, pelvic floor, electromagnetic fields, sexual dysfunction, physiological, quality of life

## Abstract

**Background and objectives**: Urinary incontinence (UI) frequently coexists with female sexual dysfunction (FSD). Magnetic chair therapies—high-intensity focused electromagnetic stimulation (HIFEM) and extracorporeal magnetic innervation (ExMI)—are increasingly used for UI, but sexual outcomes are less well synthesized. We reviewed open-access clinical studies reporting Female Sexual Function Index (FSFI) and/or Pelvic Organ Prolapse/Urinary Incontinence Sexual Questionnaire (PISQ) outcomes. **Methods**: Following PRISMA 2020 and an OSF-registered protocol, we searched PubMed, Scopus, and Web of Science to 25 October 2025. Eligible studies enrolled adult women with UI, used chair-based magnetic stimulation, and reported FSFI and/or PISQ before and after treatment. Data were narratively synthesized. **Results**: Five studies (*n* ≈ 219; FSFI *n* ≈ 170; PISQ *n* = 49) met the criteria. Randomized and controlled data showed clinically relevant advantages for active therapy: FSFI between-group gains were +6.3 at 8 weeks for HIFEM+PFMT vs. PFMT and +5.63 at 14 weeks for pulsed magnetic stimulation vs. sham. Single-arm cohorts reported FSFI increases of +8.1 at 3 months and +9.4 to +10.0 by ~6–12 months. PISQ-12 improved by +3.86 at 12 weeks when magnetic stimulation was combined with optimized PFMT. UI severity also decreased (ICIQ-UI SF −9.85; 74.4% at 12 weeks; ~71–72% reduction at 9–12 months). Adverse events were uncommon and mild where reported. **Conclusions**: Across heterogeneous designs, chair-based magnetic stimulation is associated with meaningful improvements in sexual function and continence in women with UI, with signals that combining stimulation with PFMT may enhance benefits. Standardized, longer-term trials centered on FSFI/PISQ are warranted.

## 1. Introduction

Urinary incontinence (UI) is common and erodes quality of life across physical, psychological, and relational domains; it also frequently coexists with female sexual dysfunction (FSD) via pathways that include pelvic floor weakness, dyspareunia, and fear of coital leakage [1]. Observational and review data consistently show lower sexual desire, arousal, and satisfaction among women with UI versus continent peers and highlight the specific burden of coital incontinence on intimacy and relationship dynamics [1,2]. Sexual function is typically quantified with the Female Sexual Function Index (FSFI), which profiles desire, arousal, lubrication, orgasm, satisfaction, and pain [3], and with condition-specific tools developed for pelvic floor disorders, notably the Pelvic Organ Prolapse/Urinary Incontinence Sexual Questionnaire (PISQ) family [4].

Chair-based magnetic stimulation is delivered either as extracorporeal magnetic innervation (ExMI) or as higher-output high-intensity focused electromagnetic stimulation (HIFEM). Both approaches induce rapid, painless depolarization of motor nerves and repeated supramaximal contractions of the pelvic floor musculature while the patient remains clothed and uninstrumented [5]. Randomized and controlled studies suggest short-term improvements in pelvic floor activation and continence with generally favorable safety profiles (transient soreness/paresthesias most commonly reported) [5,6,7,8].

By repeatedly recruiting pelvic floor and peri-vaginal musculature, magnetic stimulation may increase muscle bulk and endurance, enhance urethral closure pressure, and improve vaginal support and perceived tightness [9,10,11,12,13]. These neuromuscular adaptations can reduce coital leakage and urgency, increase genital blood flow, and diminish pain, thereby improving sexual desire, arousal, orgasm, satisfaction, and confidence [14,15,16,17,18,19,20,21,22,23]. Observational and interventional data link pelvic floor muscle strength with sexual function in postmenopausal women, and randomized trials show that structured pelvic floor muscle training improves multiple FSFI domains [14,22,23].

Contemporary device generations emphasize higher inductive flux densities and more focused fields intended to recruit deeper motor units and improve training efficiency compared with earlier ExMI systems [5,7,8]. For the purposes of this review, we use the term high-intensity focused electromagnetic stimulation (HIFEM), often referred to as functional magnetic stimulation (FMS), to denote newer high-output chair systems such as those used in the multicenter HIFEM cohorts [19,20]; “extracorporeal magnetic innervation” (ExMI) to describe earlier, lower-intensity chairs that generate broader pelvic fields; and “pulsed magnetic stimulation” for protocols delivering intermittent magnetic pulses with lower flux density and frequency, as in the sham-controlled trial by González-Isaza et al. [18].

While UI symptom scales such as the International Consultation on Incontinence Questionnaire—Urinary Incontinence Short Form, ICIQ-UI SF, capture frequency, volume, and bother, sexual quality-of-life endpoints are pivotal for patient-centered care—particularly in peri- and postmenopausal populations where genitourinary syndrome of menopause (GSM) amplifies dyspareunia and arousal problems [9,10,11,12]. The ICIQ-UI SF is a widely validated, brief measure of UI severity and impact suitable for trials and clinic workflows [9], and the Incontinence Impact Questionnaire short form (IIQ-7) complements it by indexing condition-specific quality-of-life impact [10]. GSM reviews underscore how vulvovaginal atrophy, dryness, and pain compound continence-related avoidance, reinforcing the rationale for trials that include FSFI/PISQ outcomes alongside UI metrics [11,12].

Across small trials and cohorts, magnetic stimulation reduces UI symptom burden, but sexual function outcomes have historically been reported piecemeal or as secondary findings without consistent prespecification [8]. A 2023 systematic review of ExMI for female UI synthesized improvements in leakage and pad test outcomes but did not center sexual endpoints, highlighting the need for focused evidence maps on FSFI/PISQ response [8]. More recently, randomized and controlled studies comparing focused magnetic stimulation (FMS/HIFEM) with pelvic floor muscle training (PFMT) have begun to clarify comparative efficacy, though sexual outcomes remain variably collected [13].

In the past 2–3 years, trials have directly measured sexual function alongside continence. A controlled study reported significant post-treatment gains in multiple FSFI domains following ExMI-augmented pelvic floor therapy compared with comparators [14]. Notably, a randomized trial in postmenopausal women demonstrated FSFI total score improvements after HIFEM combined with structured PFMT, suggesting synergy between neuromuscular stimulation and behavioral retraining [15]. Together, these developments support a focused synthesis on the magnitude and durability of FSFI/PISQ benefits with magnetic chair therapy and on how protocol parameters (field intensity, duty cycle, session dosing) and comparators (PFMT, sham) modulate effect sizes [14,15].

We therefore systematically review open-access clinical studies of chair-based magnetic pelvic floor stimulation that report FSFI and/or PISQ outcomes in women with UI, appraising device parameters, comparators, effect magnitudes for sexual function, and safety signals to inform patient-centered adoption and future trial design.

## 2. Materials and Methods

### 2.1. Protocol, Registration, and Eligibility

This review adhered to the Preferred Reporting Items for Systematic Reviews and Meta-Analyses 2020 (PRISMA 2020) guidance [16]. A completed PRISMA checklist and flow diagram will be provided as Supplementary Materials. The protocol was registered on the Open Science Framework with the registration code osf.io/p2r5y.

We included clinical studies enrolling adult women (≥18 years) with stress, mixed, or urgency urinary incontinence, or pelvic floor dysfunction with documented urinary incontinence, that evaluated noninvasive, chair-based pelvic floor magnetic stimulation (high-intensity focused electromagnetic stimulation or extracorporeal magnetic innervation) and reported sexual function outcomes using the Female Sexual Function Index and/or the Pelvic Organ Prolapse/Urinary Incontinence Sexual Questionnaire (PISQ-12 or PISQ-IR) at baseline and at least one post-treatment time point. Eligible designs were randomized controlled trials and prospective or retrospective cohorts/series with sample sizes of at least ten participants.

Articles were required to be full text, open access, and in English to ensure transparent verification of data extraction by readers, to allow clinicians and patients unrestricted access to the underlying reports, and to facilitate independent replication of our synthesis. We recognize that this decision may have excluded some paywalled or non-English studies and may therefore introduce selection bias. We excluded non-clinical studies; electrical stimulation with internal probes without magnetic stimulation; surgical, laser, or pharmacologic interventions without a magnetic stimulation arm; case reports or series with fewer than ten participants; reviews, editorials, and conference abstracts without full texts; paywalled or abstract-only items; and studies lacking FSFI or PISQ outcomes. For mixed cohorts, only data relevant to women with urinary incontinence were considered, and in multimodal interventions, inclusion required that magnetic stimulation was a core component with sexual function outcomes attributable to, or clearly separable for, that pathway.

The prespecified PICO framing was the following: among adult women with urinary incontinence (population), does noninvasive chair-based pelvic floor magnetic stimulation, alone or as an adjunct to conservative care (intervention), compared with sham, pelvic floor muscle training, or other conservative approaches, or the participant’s own baseline in pre–post designs (comparators), improve sexual function as measured by the FSFI and/or PISQ (outcomes).

### 2.2. Information Sources and Search Strategies

We searched PubMed/MEDLINE, Scopus, and Web of Science Core Collection from database inception through 25 October 2025, without date limits. To enhance completeness, we screened the reference lists of records taken to full-text review and relevant topical reviews. The grey literature and trial registries were not systematically searched because inclusion required open-access, full-text manuscripts suitable for verifiable extraction.

Our search strategies combined controlled vocabulary, where available, with free-text terms representing three concept blocks: magnetic chair therapies, urinary incontinence phenotypes, and sexual function instruments. In PubMed/MEDLINE, we searched using the following search string: ((„high-intensity focused electromagnetic“[Title/Abstract] OR HIFEM[Title/Abstract] OR „functional magnetic stimulation“[Title/Abstract] OR „extracorporeal magnetic innervation“[Title/Abstract] OR ExMI[Title/Abstract] OR „pulsed magnetic stimulation“[Title/Abstract] OR „magnetic chair“[Title/Abstract] OR emsella[Title/Abstract] OR neocontrol[Title/Abstract]) AND („urinary incontinence“[MeSH Terms] OR „urinary incontinence“[Title/Abstract] OR „stress urinary incontinence“[Title/Abstract] OR SUI[Title/Abstract] OR „mixed urinary incontinence“[Title/Abstract] OR „overactive bladder“[Title/Abstract] OR OAB[Title/Abstract] OR „pelvic floor“[Title/Abstract]) AND („Female Sexual Function Index“[Title/Abstract] OR FSFI[Title/Abstract] OR PISQ[Title/Abstract] OR „Pelvic Organ Prolapse/Urinary Incontinence Sexual Questionnaire“[Title/Abstract] OR „PISQ-12“[Title/Abstract] OR „PISQ-IR“[Title/Abstract])) AND (english[lang]) AND (female[MeSH Terms]) AND („humans“[MeSH Terms]) AND („journal article“[Publication Type]) AND („2000/01/01“[Date—Publication]: „2025/10/25“[Date—Publication]).

In Scopus, we queried titles, abstracts, and keywords for the same three concept blocks; specifically, we searched for “high-intensity focused electromagnetic,” HIFEM, “extracorporeal magnetic innervation,” ExMI, “pulsed magnetic stimulation,” “magnetic chair,” Emsella, “BTL Emsella,” or NeoControl, in conjunction with “urinary incontinence,” “stress urinary incontinence,” SUI, “mixed urinary incontinence,” “overactive bladder,” OAB, or “pelvic floor,” and in conjunction with “Female Sexual Function Index,” FSFI, PISQ, “PISQ-12,” or “PISQ-IR,” then refined to English-language research articles and applied the Open Access filter; any records lacking true open-access full texts were excluded at full-text screening. In Web of Science Core Collection, we performed a Topic search using the same three concept blocks joined with AND, refined to English, document type Article, and excluded items without accessible open-access full texts during full-text screening. All searches were last updated on 25 October 2025.

### 2.3. Selection Process

**Figure 1:**
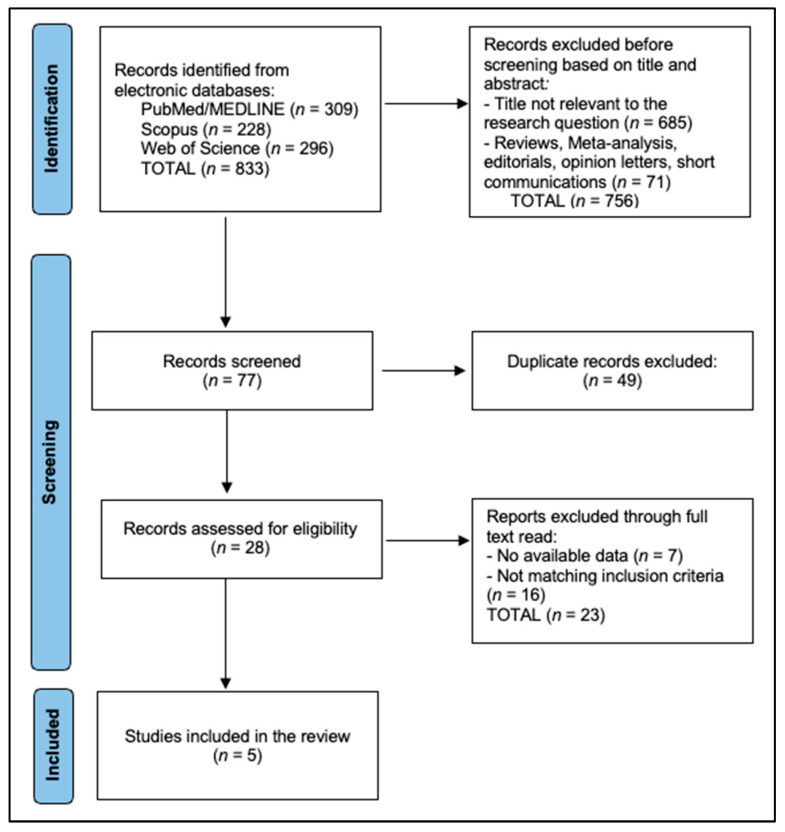
PRISMA flowchart diagram.

Search results were exported and merged in a reference manager, and duplicates were removed. Two reviewers independently screened titles and abstracts against the eligibility criteria, retrieved potentially eligible full texts, and performed independent full-text assessments. Disagreements at either stage were resolved through discussion, with a third reviewer available for arbitration when necessary. Reasons for exclusion at the full-text stage were recorded and summarized in the PRISMA flow diagram (Figure 1).

Following database searches (PubMed/MEDLINE *n* = 309, Scopus *n* = 228, Web of Science *n* = 296; total *n* = 833), we excluded 756 records at title/abstract level for irrelevance to the research question (*n* = 685) or ineligible publication type (reviews, meta-analyses, editorials, opinion letters, short communications; *n* = 71). The remaining 77 records were screened, during which 49 duplicates were removed, leaving 28 reports for full-text assessment. Of these, 23 were excluded following full-text review—7 for having no available/extractable data and 16 for not meeting the inclusion criteria—resulting in 5 studies being included in the review [17,18,19,20,21]. 

### 2.4. Data Collection Process

We captured study design and setting; sample size; participant characteristics including age and menopausal status when available; urinary incontinence subtype; intervention characteristics including device or platform and dosing (session duration, frequency, number, and total exposure); comparator details when applicable; follow-up timing; sexual function instruments and summary statistics at baseline and follow-up, change scores, secondary continence outcomes such as ICIQ-UI SF, IIQ-7, pad tests, and pelvic floor strength measures; safety outcomes and withdrawals; and funding or conflicts of interest. When unavailable, variables were coded as not reported.

The primary data items were FSFI total score and, when available, domain scores, as well as PISQ outcomes (PISQ-12 or PISQ-IR). Secondary data items included continence measures (ICIQ-UI SF and IIQ-7), pad test results, pelvic floor strength metrics, adverse events, and attrition. We prespecified the extraction of time points closest to the principal post-treatment assessment for each study, alongside baseline values, to enable pre–post and, where applicable, between-group comparisons. Where studies reported multiple follow-ups, we preferentially extracted the earliest post-treatment time point designated as primary by the authors; additional time points were recorded when available.

Given the small number of heterogeneous studies and incomplete reporting of variance estimates, we prespecified that any quantitative synthesis would be descriptive.

### 2.5. Quantitative Synthesis

We considered conducting a random-effects meta-analysis of FSFI total score change and constructing a forest plot of standardized mean differences. However, the included studies differed substantially in population (menopausal status, UI subtype), intervention protocols (HIFEM vs. pulsed magnetic stimulation vs. magnetic stimulation combined with optimized PFMT), comparator groups, and follow-up timing, and several lacked standard deviations for change scores. In view of these limitations and the risk of generating misleading pooled estimates from sparse and heterogeneous data, we restricted our quantitative synthesis to descriptive presentation of study-level mean changes and between-group differences.

In this review, we use ‘chair-based magnetic pelvic floor stimulation’ as an umbrella term that encompasses ExMI, HIFEM/functional magnetic stimulation, and lower-intensity pulsed magnetic stimulation protocols.

### 2.6. Risk of Bias and Certainty of Evidence

Risk of bias was assessed independently by two reviewers using the RoB 2 tool for randomized trials and ROBINS-I for nonrandomized studies. Evaluations considered the randomization process or, for nonrandomized designs, confounding and selection; deviations from intended interventions; completeness of outcome data; outcome measurement including instrument validity and feasibility of blinding; and selection of the reported result. Discrepancies were resolved by consensus. We recorded study funding sources and any disclosed relationships with device manufacturers to contextualize judgments.

Both randomized trials showed at least “some concerns” in one or more RoB 2 domains, primarily related to lack of blinding of participants and personnel and incomplete reporting of adverse events [17,18]. The retrospective and prospective cohorts were judged at moderate to serious risk of bias in ROBINS-I, mainly because of confounding, nonrandom allocation to intervention arms, and limited reporting of co-interventions and attrition [19,20,21]. Across designs, selective reporting of sexual outcomes and incomplete adverse event reporting were common. No study was judged to be at overall low risk of bias across all domains. In addition, we used the GRADE framework to summarize the overall certainty of evidence for key outcomes (FSFI total score, PISQ-12, and ICIQ-UI SF). GRADE ratings (high, moderate, low, very low).

Using GRADE, the certainty of evidence for improvement in the FSFI total score with chair-based magnetic stimulation was rated as very low, reflecting downgrades for risk of bias, inconsistency between studies (ranging from null to large effects), imprecision (small samples, wide confidence intervals, and incomplete variance reporting), and suspected publication bias. Certainty for continence improvement (ICIQ-UI SF) was rated as low because of similar concerns but more consistent direction of effect, and certainty for PISQ-12 improvement was rated as very low given that only a single retrospective cohort contributed data [17,18,19,20,21], as presented in Table 1 and Table 2.

## 3. Results

Across five open-access clinical studies [17,18,19,20,21], designs ranged from randomized controlled trials to prospective and retrospective cohorts, covering stress urinary incontinence (SUI), mixed UI, or pelvic floor dysfunction with documented UI, and spanned multiple settings (US/EU, Colombia, China). In Elgayar 2024 [17], postmenopausal women received high-intensity focused electromagnetic stimulation (HIFEM) combined with pelvic floor muscle training (PFMT) versus PFMT alone, with sexual function assessed at 8 weeks using the FSFI. González-Isaza 2022 [18] randomized women with SUI to pulsed magnetic stimulation or sham and evaluated FSFI at 14 weeks. The multicenter cohort by Hlavinka 2019 [19] delivered six chair-based HIFEM sessions over three weeks and followed participants for 1–3 months with FSFI outcomes. Evans 2023 [20] used a standardized 6–8 HIFEM session regimen and collected both FSFI and PISQ-12 at roughly 6 months. Finally, Wang 2022 [21] retrospectively compared pelvic floor magnetic stimulation alone versus in combination with optimized PFMT in moderate SUI, recording PISQ-12 at 6–12 weeks, as seen in Table 3.

Table 4 synthesizes FSFI/PISQ changes by reporting baseline and follow-up means, mean changes (Δ), and 95% confidence intervals where available. Across studies, FSFI total score mean changes ranged from −1.2 points (no benefit in the pulsed magnetic stimulation arm at 14 weeks) to +10.2 points at 3-month follow-up in the single-arm HIFEM cohort, with between-group FSFI advantages of +5.63 and +6.3 points in favor of active magnetic stimulation versus sham and PFMT alone, respectively [17,18,19]. PISQ-12 mean scores improved by +3.86 points at 12 weeks when magnetic stimulation was combined with optimized PFMT [21].

Where FSFI totals were reported, mean post-treatment scores in the HIFEM multicenter cohorts crossed the validated clinical cutoff of 26.55 points for differentiating women with and without sexual dysfunction [22,23,24,25,26,27,28,29]. Specifically, the single-arm cohort by Hlavinka 2019 improved from a mean FSFI total of 20.06 at baseline to 30.29 at 3 months, and the prospective multicenter study by Evans 2023 reported mean FSFI gains of approximately +9.4 to +10.0 points from baseline values already near the cutoff [19,20,29]. In contrast, post-treatment FSFI means in Elgayar 2024 (16.04 → 24.00) and the pulsed magnetic stimulation trial by González-Isaza 2022 (24.39 → 23.19 in the active arm) remained below 26.55, indicating that statistically significant or numerically modest changes may not always translate into the resolution of sexual dysfunction at the group level [17,18,29].

**Figure 2:**
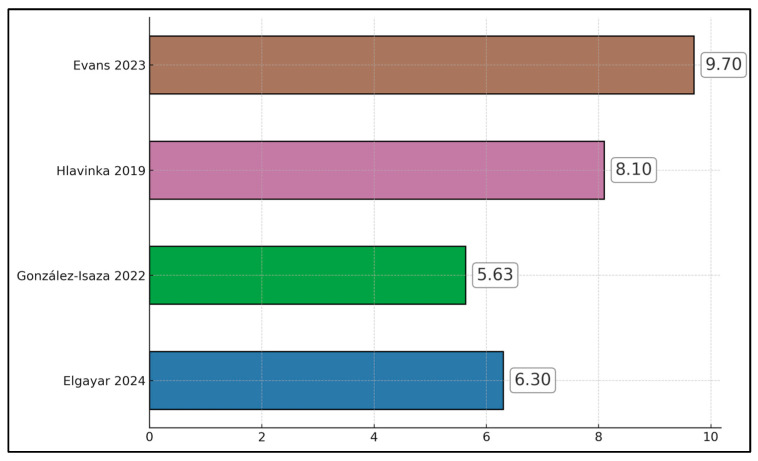
Bar chart of FSFI total score change (post/last follow-up minus baseline) [17,18,19,20].

Three of four studies reported marked improvements in sexual function: Hlavinka 2019 (+10.23 FSFI points from 20.06 → 30.29 at 3-month follow-up) [19]; Evans 2023 (+9.4 FSFI points after final treatment; subgroup A) [20]; and Elgayar 2024 (+7.96 FSFI points in the HIFEM arm from 16.04 → 24.00) [17]. In contrast, González-Isaza 2022 did not show an FSFI gain in the treatment arm at 14 weeks (24.39 → 23.19; −1.20 points), although that RCT emphasized continence benefits (ICIQ-UI SF) and pelvic floor strength gains rather than FSFI [18], as presented in Figure 2.

Table 5 integrates continence severity (ICIQ-UI SF), sexual function trajectories (FSFI, PISQ-12), objective leakage, pelvic floor strength, and safety reporting across studies. Wang 2022 [21] showed the largest continence improvement with ICIQ-UI SF 13.24 → 3.39 (Δ − 9.85; 74.4% reduction) at 12 weeks, concurrent PISQ-12 28.61 → 32.47 (Δ + 3.86), a 1 h pad test drop 6.4 → 1.6 g, and EMG phasic/tonic rises 24.3 → 41.2 μV/19.2 → 38.9 μV, indicating both symptom and physiological gains. In the multicenter series, Evans 2023 [20] reported ~71–72% ICIQ-UI SF improvement at 9–12 months (largest mean decreases 8.6–9.3 points) and maximal FSFI gains +9.4 to +10.0, with domain-level improvements (desire, arousal, lubrication, satisfaction) and PISQ-12 enhancement noted. The RCT of González-Isaza 2022 [18] recorded ICIQ-UI SF 11.41 → 7.13 (Δ − 4.28) and Oxford scale strength 1.68 → 2.81, though FSFI changed 24.39 → 23.19 (Δ − 1.20) by 14 weeks. Single-arm cohorts demonstrated robust FSFI gains: Hlavinka 2019 [19] 20.06 → 30.69 post-treatment and → 30.29 at 3 months (Δ + 10.23), while Elgayar 2024 [17] reported FSFI 16.04 → 24.00 (Δ + 7.96) at the end of treatment, alongside a PFMT-outperforming rise in pelvic floor strength of 2.7 ± 1.4 → 3.2 ± 1.2. Adverse events were not reported across rows that provided outcomes (NR).

**Figure 3:**
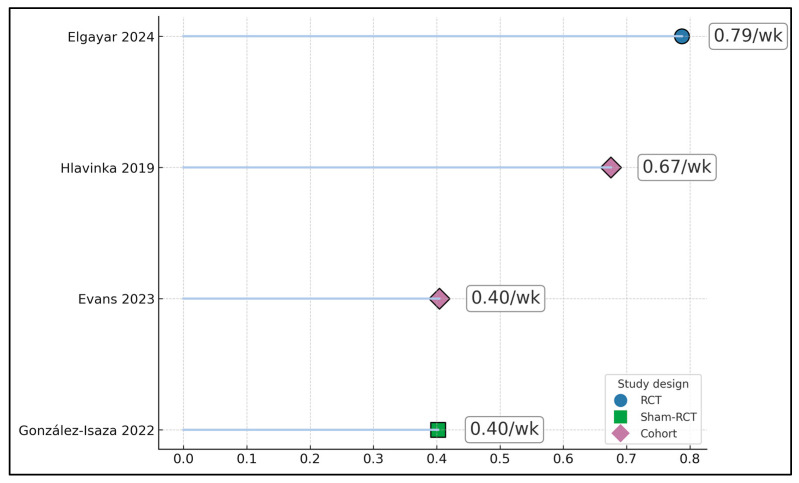
Scatter of percent reduction in ICIQ-UI SF (baseline → last follow-up) [17,18,19,20].

The strongest continence gains (ICIQ-UI SF reduction) were observed in the retrospective cohort with combined therapy (Wang 2022, 13.24 → 3.39; 74.4% reduction at 12 weeks) [21]. The multicenter prospective study (Evans 2023) reported ~72% improvement at 9–12 months (largest mean decreases 8.6–9.3 ICIQ-SF points) [20]. The sham-controlled RCT (González-Isaza 2022) still achieved a 37.5% reduction in ICIQ-SF in the treatment arm (11.41 → 7.13) at 14 weeks [18], as seen in Figure 3.

## 4. Discussion

### 4.1. Summary of Evidence

This systematic review of five open-access clinical studies suggests that chair-based magnetic pelvic floor stimulation is associated with clinically meaningful improvements in sexual function and urinary symptoms in women with urinary incontinence [17,18,19,20,21]. However, the certainty of evidence is low to very low for sexual endpoints and low for continence outcomes because of small samples, heterogeneous designs, and risk-of-bias concerns.

Regarding the similar literature, in a randomized trial in women with overactive bladder, supervised pelvic floor muscle training (PFMT) improved the FSFI total and several subscales versus the control, indicating that neuromuscular conditioning alone can translate into meaningful sexual gains [22]. Moreover, a 2024 meta-analysis of randomized trials concluded that PFMT increases the FSFI total score by a pooled +7.67 points (95% CI 0.77–14.57), with improvements in arousal, orgasm, satisfaction, and pain—albeit with low certainty because of heterogeneity [23]. These data support our observation that HIFEM/ExMI may augment sexual outcomes, and they reinforce the biologic plausibility of additive or synergistic effects when magnetic stimulation is paired with structured PFMT.

Positioning magnetic stimulation within the spectrum of conservative continence therapies also helps contextualize effect sizes. A 2024 network meta-analysis of 31 RCTs comparing non-surgical options for stress urinary incontinence ranked magnetic stimulation mid-pack for improving ICIQ-UI SF, with higher rankings for electrical stimulation–based approaches; nevertheless, magnetic stimulation remained an effective option overall [29]. Head-to-head trial data likewise suggest functional magnetic stimulation can achieve continence and quality-of-life benefits comparable to PFMT in women with SUI, supporting our inference that protocolized magnetic regimens are clinically relevant alternatives or adjuncts when adherence or access to supervised PFMT is limited [25].

Device-specific parameters may partly explain between-study variability in sexual outcomes. Classical ExMI chairs generate lower-intensity, broader magnetic fields with longer pulse trains, whereas HIFEM/FMS platforms deliver higher-intensity, more focused fields with rapid pulse sequences designed to elicit repeated supramaximal contractions of the levator ani and peri-vaginal musculature [5,7,13,19,20]. Pulsed magnetic protocols such as those used by González-Isaza et al. [18] occupy an intermediate position, with lower flux density and intermittent activation. It is therefore plausible that HIFEM-type regimens, particularly when combined with structured PFMT, may produce larger gains in pelvic floor strength and sexual function than lower-intensity or less standardized protocols, as suggested by the larger FSFI improvements observed in the multicenter HIFEM cohorts compared with the pulsed stimulation RCT [18,19,20,21]. However, the small number of studies and incomplete reporting of technical parameters prevent firm conclusions, and direct head-to-head comparisons of ExMI versus HIFEM using harmonized dosing and prespecified sexual endpoints are needed.

A key driver of inconsistency across sexual endpoints is measurement. Many women with pelvic floor disorders are not sexually active at baseline. In such cohorts, the PISQ-IR is more responsive than the FSFI for not-sexually active participants, whereas both questionnaires are responsive among sexually active women [24]. Interpretation of post-treatment FSFI values also benefits from anchoring to diagnostic thresholds. Cross-validation work established 26.55 as an optimal FSFI total cutoff to distinguish women with and without sexual dysfunction [29]. Pre–post gains that remain below this threshold may therefore be statistically significant but not clinically meaningful for some patients.

Two clinical contexts deserve emphasis because they modulate sexual outcomes independent of continence reduction. Firstly, coital incontinence (CI) is highly prevalent and tightly coupled to both SUI and OAB phenotypes; a 2024 multicenter analysis of 4843 women found CI in 42% of those with urinary incontinence, with moderate correlations to both SUI and OAB symptom scores [28]. Secondly, genitourinary syndrome of menopause (GSM) and vulvovaginal atrophy contribute dyspareunia and lubrication problems that neural/muscular stimulation may not directly address. Meta-analyses of energy-based vaginal therapies report mixed and often heterogeneous effects on sexual function—improvements are possible, but certainty is limited and generalizability to magnetic chair outcomes is uncertain [27].

Interpretation of FSFI and PISQ trajectories is further complicated by multiple potential confounders that were incompletely captured across studies. Baseline sexual activity status, relationship stability, use of systemic or local hormone therapy, concomitant psychosexual counseling, analgesic and antidepressant use, and secular changes in mood or chronic pain could all influence sexual desire, arousal, and satisfaction independently of pelvic floor interventions. In addition, variability in the intensity and adherence to concomitant PFMT, as well as unreported GSM-directed treatments, may have contributed to between-study differences in sexual outcomes.

Finally, durability and dosing require standardization. The network meta-analysis highlights that comparative rankings are sensitive to protocol heterogeneity (session number, frequency, field characteristics) and to choice of outcomes and time points [29]. Our review’s strongest mid-to-long-term FSFI gains appeared in structured multi-session programs, consistent with broader rehabilitation principles and with trials showing that more intensive supervised regimens yield larger sexual function benefits [23]. We recommend that forthcoming trials adopt sham-controlled designs where feasible; register protocols prospectively; prespecify sexual outcomes (FSFI total and domains, PISQ-IR, and, when appropriate, sexual distress scales) as primary or co-primary endpoints; clearly define and standardize magnetic stimulation parameters (flux density, frequency, duty cycle, and number of sessions); and report both mean changes and responder proportions crossing clinically meaningful thresholds such as the FSFI 26.55 cutoff [29].

For women with stress or mixed UI—particularly peri- and postmenopausal patients—magnetic chair therapy offers a noninvasive, time-efficient option that can be delivered while clothed and without intravaginal instrumentation. Clinicians can reasonably expect short-term FSFI total score gains in the range of ~6–10 points and concurrent reductions in ICIQ-UI SF of ~40–75% in responsive cohorts. Integrating stimulation with structured pelvic floor muscle training appears to augment sexual function (PISQ-12 +3.86) and continence effects, supporting a combined behavioral–neuromodulatory approach. Routine collection of FSFI (total and domains) and/or PISQ-12 at baseline and at 8–12 weeks, with follow-up at 6–12 months, can help identify responders, guide maintenance schedules, and align outcomes with patient-centered goals.

### 4.2. Limitations

The evidence base is limited by small samples, heterogeneous devices and dosing (session duration and number of sessions, field intensity, duty cycle), and variable prespecification of sexual endpoints. Only open-access, English-language studies were included, which likely excluded some paywalled or non-English trials and may bias the available evidence toward industry-sponsored or selectively disseminated reports. Follow-up beyond 6–12 months is sparse, and adverse event reporting was inconsistent. Between-study differences in menopausal status, UI phenotype, and concomitant PFMT complicate effect attribution. One RCT did not show FSFI improvement despite continence gains, underscoring potential discordance between symptom and sexual outcomes. Finally, meta-analysis was not attempted because of between-study heterogeneity and incomplete variance reporting.

Finally, several of the included studies used proprietary commercial HIFEM systems, and the multicenter cohorts appear to have been closely aligned with device implementation in routine practice [19,20].

## 5. Conclusions

Current open-access clinical evidence suggests that chair-based magnetic pelvic floor stimulation improves sexual function and continence in women with UI, with the largest and most consistent gains observed when therapy is paired with pelvic floor muscle training. Safety appears favorable. Future adequately powered, sham-controlled trials should standardize FSFI/PISQ collection, report domain-level trajectories, delineate optimal dosing, and assess durability to 12–24 months.

## Figures and Tables

**Table 1:** Risk-of-bias assessment for randomized controlled trials (RoB 2).

Study (Year)	Randomization Process	Deviations from Intended Interventions	Missing Outcome Data	Measurement of the Outcome	Selection of the Reported Result	Overall RoB 2 Judgement
Elgayar 2024 [17]	Some concerns—randomization described but allocation concealment and baseline balance for sexual activity/FSFI domains not fully reported.	Some concerns—participants and personnel were not clearly blinded; PFMT intensity and other co-interventions may have differed between groups.	Low risk—no major differential loss to follow-up for FSFI reported; analysis appears to include most randomized women.	Some concerns—FSFI is self-reported and outcome assessors were not blinded; knowledge of group assignment could have influenced responses.	Some concerns—protocol not publicly available; selective reporting of adverse events and incomplete detail on prespecified sexual outcomes.	Some concerns
González-Isaza 2022 [18]	Low risk—random allocation and sham control described; baseline characteristics broadly similar between groups.	Some concerns—sham protocol described but not all details on adherence and co-interventions; possibility of unblinding due to treatment sensations.	Low risk—follow-up at 14 weeks largely complete; no evidence of strongly differential attrition between arms.	Some concerns—FSFI is self-reported; blinding of outcome assessment not explicitly confirmed, and participants may have inferred allocation.	Some concerns—no registered protocol; FSFI and adverse events reported incompletely relative to continence outcomes.	Some concerns

RoB 2, revised Cochrane risk-of-bias tool for randomized trials; FSFI, Female Sexual Function Index; PFMT, pelvic floor muscle training.

**Table 2:** Risk-of-bias assessment for nonrandomized studies (ROBINS-I).

Study (Year)	Confounding	Selection of Participants into the Study	Classification of Interventions	Deviations from Intended Interventions	Missing Data	Measurement of Outcomes	Selection of the Reported Result	Overall ROBINS-I Judgment
Hlavinka 2019 [19]	Serious—single-arm cohort without control group; no adjustment for age, menopausal status, baseline sexual function, or concomitant therapies.	Moderate—convenience sample of treatment-seeking women; eligibility criteria only partially described.	Low—HIFEM regimen clearly defined and consistently applied.	Moderate—no blinding; co-interventions (PFMT, hormonal treatments) and adherence not systematically captured.	Moderate—follow-up to 3 months with limited reporting of attrition; unclear whether missing FSFI data were related to outcomes.	Moderate—FSFI is validated, but self-reported with unblinded participants and no independent outcome assessor.	Serious—no pre-registered protocol; sexual outcomes selectively emphasized, adverse events sparsely reported.	Serious
Evans 2023 [20]	Serious—prospective multicenter cohort without control group; substantial potential for confounding by indication, center-level practice, PFMT use, hormone therapy, and baseline sexual activity.	Moderate—participants were women undergoing HIFEM in routine practice; selection mechanisms only partly described.	Low—HIFEM treatment defined (6–8 sessions) with consistent classification of exposure.	Moderate—no blinding; concomitant conservative measures and lifestyle changes not controlled or fully reported.	Serious—9–12-month follow-up with incomplete and poorly described attrition; unclear handling of missing FSFI/PISQ-12 data.	Moderate—FSFI and PISQ-12 are validated, but self-reported; no blinding of participants or assessors.	Serious—lack of protocol; partial reporting of domain-level sexual outcomes and adverse events; potential for selective emphasis on favorable results.	Serious
Wang 2022 [21]	Serious—retrospective comparison of magnetic stimulation alone vs. magnetic + optimized PFMT; allocation strongly influenced by clinical judgment and patient preference; limited adjustment for confounders.	Moderate—inclusion based on treated moderate SUI cases; reasons for entering each treatment pathway are incompletely described.	Low—interventions (magnetic alone vs. magnetic + PFMT) clearly defined in records.	Moderate—adherence to PFMT and other conservative therapies not systematically captured; no blinding.	Serious—only a subset (*n* = 49 of 95) had analyzable PISQ-12 data; handling of missing sexual function data not clearly reported.	Moderate—PISQ-12 and ICIQ-UI SF are validated; outcomes self-reported with awareness of treatment; no blinded assessment.	Serious—no prespecified protocol; sexual outcomes reported for a subset of the cohort; limited adverse-event reporting.	Serious

ROBINS-I, Risk of Bias In Non-randomized Studies of Interventions; HIFEM, high-intensity focused electromagnetic stimulation; PFMT, pelvic floor muscle training; FSFI, Female Sexual Function Index; PISQ-12, Pelvic Organ Prolapse/Urinary Incontinence Sexual Questionnaire-12; ICIQ-UI SF, International Consultation on Incontinence Questionnaire—Urinary Incontinence Short Form.

**Table 3:** Characteristics of included studies.

Study (Year)	Country/Setting	Design and Population	UI Subtype	Device/Regimen	Comparator	Follow-Up	Sexual Instrument
Elgayar 2024 [17]	NR	RCT, postmenopausal women	NR (pelvic floor dysfunction; UI status NR)	HIFEM + PFMT; session details NR in abstract	PFMT alone	8 weeks	FSFI
González-Isaza 2022 [18]	Colombia	Randomized, sham-controlled SUI	SUI	Pulsed magnetic stimulation; parameters per protocol	Simulation (sham)	14 weeks	FSFI
Hlavinka 2019 [19]	Multicenter (US/EU)	Prospective multicenter cohort	Mixed UI/FSD	HIFEM protocol (chair); 6 sessions over 3 weeks (typical)	None (single arm)	1–3 months	FSFI
Evans 2023 [20]	Multicenter	Prospective multicenter	UI + FSD	HIFEM; standardized 6–8 sessions	None (single arm)	6 months	FSFI, PISQ-12
Wang 2022 [21]	China	Retrospective cohort; *n* ≈ 95	Moderate SUI	Pelvic floor magnetic stimulation ± optimized PFMT	Active comparator (magnetic alone)	6–12 weeks	PISQ-12

UI, urinary incontinence; SUI, stress urinary incontinence; HIFEM, high-intensity focused electromagnetic stimulation; PFMT, pelvic floor muscle training; FSFI, Female Sexual Function Index; PISQ-12, Pelvic Organ Prolapse/Urinary Incontinence Sexual Questionnaire-12; RCT, randomized controlled trial; NR, not reported.

**Table 4:** Sexual function outcomes (FSFI/PISQ): baseline and follow-up means, mean changes (Δ), and 95% confidence intervals where reported.

Study	Instrument	Baseline Mean (SD)/Time	Change (Δ)/Between-Group Effect	*p*-Value
Elgayar 2024 [17]	FSFI total	16.04	Follow-up 24.00 at 8 weeks; Δ + 7.96 within HIFEM+PFMT; between-group FSFI advantage +6.3 points vs. PFMT alone	<0.001
González-Isaza 2022 [18]	FSFI total	24.39	Follow-up 23.19 at 14 weeks in active arm; Δ − 1.20; between-group FSFI difference +5.63 in favor of active vs. sham	<0.05
Hlavinka 2019 [19]	FSFI total	20.06	Follow-up 30.69 post-treatment and 30.29 at 3 months; Δ + 10.23 at 3 months	<0.001
Evans 2023 [20]	FSFI total; PISQ-12	NR	Approximate mean increases +9.4 to +10.0 at ~6 months; exact baseline and follow-up means NR; PISQ-12 improved	NR
Wang 2022 [21]	PISQ-12 (direction per instrument used in paper)	28.61	Follow-up 32.47 at 12 weeks in magnetic + optimized PFMT arm; Δ + 3.86	<0.05

FSFI, Female Sexual Function Index (0–36); PISQ-12, Pelvic Organ Prolapse/Urinary Incontinence Sexual Questionnaire-12; Δ, change; SD, standard deviation; CI, confidence interval; w, weeks; mo, months; NR, not reported.

**Table 5:** Safety and continence/sexual outcomes.

Study (Year)	N	Follow-Up Time Point Used	ICIQ-UI SF (Baseline → Follow-Up)	FSFI Total (Baseline → Follow-Up)	PISQ-12 (Baseline → Follow-Up)	1 hr Pad Test (g) Baseline → Follow-Up	Oxford/EMG or PFM Strength	Adverse Events
Elgayar 2024 [17]	62	End of treatment (8 sessions)	NR	16.04 → 24.00 (Δ + 7.96)	NR	NR	PFM 2.7 ± 1.4 → 3.2 ± 1.2; combined regimen outperformed PFMT alone	NR
González-Isaza 2022 [18]	47	14 weeks	11.41 → 7.13 (Δ − 4.28)	24.39 → 23.19 (Δ − 1.20)	NR	NR	Oxford 1.68 ± 0.99 → 2.81 ± 0.72	NR
Hlavinka 2019 [19]	30	Post-treatment and 3-month	NR	20.06 → 30.69 post; → 30.29 at 3-mo (Δ + 10.23)	NR	NR	NR	NR
Evans 2023 [20]	31	9–12 months	Mean ICIQ-UI SF ↓ 71–72%; largest mean ↓ 8.6–9.3 points; small, nonsignificant relapse thereafter	Max FSFI gain +9.4 to +10.0 points	↑ in desire, arousal, lubrication, satisfaction subdomains reported	NR	NR	NR
Wang 2022 [21]	49 (of 95 total)	12 weeks	13.24 → 3.39 (Δ − 9.85; 74.4% reduction)	NR	28.61 → 32.47 (Δ + 3.86); emotional 7.82 → 8.78; physiological 14.37 → 17.24; partner 6.43 → 6.45 (NS)	6.4 → 1.6 g	EMG phasic 24.3 → 41.2 μV; tonic 19.2 → 38.9 μV	NR

ICIQ-UI SF, International Consultation on Incontinence Questionnaire—Urinary Incontinence Short Form; FSFI, Female Sexual Function Index; PISQ-12, Pelvic Organ Prolapse/Urinary Incontinence Sexual Questionnaire-12; PFM, pelvic floor muscle; EMG, electromyography; g, grams; μV, microvolts; NR, not reported.

## Supplementary Materials

The following are available online at https://www.mdpi.com/article/10.3390/jcm14238496/s1, File S1: RISMA 2020 Checklist.
